# Frequency-dependent modulation of evoked potentials during GPi-DBS in pediatric dystonia

**DOI:** 10.1038/s41598-025-18436-6

**Published:** 2025-09-29

**Authors:** Rahil Soroushmojdehi, Jessica S. L. Vidmark, S. Alireza Seyyed Mousavi, Sumiko Abe, Terence D. Sanger

**Affiliations:** 1 Department of Electrical Engineering and Computer Science, University of California, Irvine, CA USA; 2 Department of Biomedical Engineering, University of California, Irvine, CA USA; 3 Department of Neurology, Children’s Hospital of Orange County, Orange, CA USA

**Keywords:** Deep brain stimulation (DBS), Evoked potentials (EP), Pediatric dystonia, Globus pallidus internus (GPi), Stimulation frequency, Electrophysiology, Neuronal physiology, Neurophysiology, Network models, Basal ganglia, Motor neuron, Neural circuits, Dystonia, Biomedical engineering

## Abstract

Deep brain stimulation (DBS) of the globus pallidus internus (GPi) is an established treatment for dystonia, yet the neurophysiological mechanisms underlying frequency-dependent effects remain poorly understood. In this study, we investigate how different GPi-DBS frequencies modulate evoked potential (EP) characteristics within the basal ganglia-thalamo-cortical network in pediatric dystonia patients. We evaluated how increasing stimulation frequency alters EP morphology, focusing on peak-to-peak amplitude (PPa), peak-to-peak duration (PPd), and time to peak (TP). This analysis was motivated by clinical observations that higher DBS frequencies often produce distinct therapeutic effects, suggesting underlying differences in how neural circuits respond to varying stimulation rates. We analyzed intracranial electrophysiological recordings from 13 pediatric and young adult dystonia patients undergoing staged DBS implantation. EPs were recorded from the GPi and subthalamic nucleus (STN) of basal ganglia, and ventral oralis (VO) nucleus of the thalamus during unilateral GPi stimulation at 55, 85, 185, and 250 Hz. EP detection and characterization were performed using an automated algorithm, and group-level analyses were conducted using linear mixed effects models to assess frequency-dependent changes across regions. Increasing stimulation frequency significantly decreased PPa and increased PPd and TP, with a clear threshold effect: 55 Hz and 85 Hz produced similar responses, while 185 Hz and 250 Hz elicited significantly greater changes. These frequency-dependent effects were most pronounced in GPi recordings, followed by STN and VO, suggesting that local circuit dynamics are more sensitive to frequency modulation. Our findings highlight the distinct neurophysiological effects of DBS frequency on EP characteristics, emphasizing the need for personalized DBS programming. The pronounced frequency-dependent modulation observed in GPi suggests that optimal stimulation parameters should be tailored to the target region rather than applying uniform settings across patients. Future studies should investigate the clinical relevance of these EP dynamics and explore their potential as biomarkers for DBS optimization in dystonia.

## Introduction

Deep brain stimulation (DBS) of the globus pallidus internus (GPi) is an effective treatment for medically refractory dystonia, providing sustained symptom relief in both pediatric and adult patients^1–8^. While clinical outcomes vary, a growing body of research suggests that the therapeutic efficacy of DBS is influenced by stimulation parameters, particularly frequency^9–12^. However, the underlying neurophysiological mechanisms through which DBS frequency modulates basal ganglia network activity are poorly understood. A deeper understanding of these mechanisms is crucial for optimizing DBS programming and improving patient outcomes.

DBS elicits evoked potentials (EPs) in local and distant brain regions, offering an electrophysiological readout of stimulation-induced network responses. While informative, the specificity and clinical significance of EPs remain under investigation. EPs recorded during DBS have been observed in the GPi, subthalamic nucleus (STN), and thalamus, with their characteristics (e.g., amplitude, latency, and duration) modulated by stimulation frequency^13–15^. Previous studies in Parkinson’s disease (PD) have shown that higher stimulation frequencies (e.g., 130 Hz and above) reduce EP amplitudes and prolong response latencies, suggesting cumulative synaptic refractoriness and inhibitory network effects^13^. Similar phenomena have been observed in dystonia, where GPi-DBS at higher frequencies suppresses pathological oscillations and alters neuronal firing patterns^14^. However, most prior studies have either focused on single stimulation frequencies or examined DBS effects in PD, leaving a gap in knowledge regarding how different DBS frequencies shape neurophysiological responses in dystonia.

The goal of this study is to systematically investigate how DBS frequency modulates EP characteristics in pediatric dystonia patients. Specifically, we analyze EPs recorded from the GPi, STN, and ventral oralis (VO) nucleus of the thalamus during unilateral GPi-DBS at 55, 85, 185, and 250 Hz. Based on prior studies, we hypothesize that increasing stimulation frequency will lead to a progressive decrease in EP amplitude and an increase in EP latency measures. Furthermore, we predict that these effects will be most pronounced in GPi recordings, with weaker modulation observed in STN and VO, reflecting regional differences in circuit integration and frequency sensitivity.

Although this study does not directly assess clinical outcomes, its findings have important implications for DBS programming in dystonia. Many patients require higher stimulation frequencies (>150 Hz) to achieve optimal symptom relief, but the ideal frequency varies across individuals^8,16,17^. Understanding how DBS frequency differentially affects neural targets could guide personalized stimulation strategies and help refine DBS settings based on electrophysiological markers rather than trial-and-error adjustments.

To test our hypotheses, we analyzed intracranial electrophysiological recordings from 13 pediatric and young adult dystonia patients undergoing staged DBS implantation. We applied an automated EP detection algorithm to quantify amplitude and latency across stimulation conditions and performed group-level analyses using mixed effects modeling to assess frequency-dependent trends. This study provides new insights into how DBS frequency shapes basal ganglia network responses, with potential implications for optimizing neuromodulation therapies in dystonia and other movement disorders.

## Materials and methods

### Study participants

Thirteen pediatric and young adult patients diagnosed with primary or secondary dystonia by a movement disorder specialist (T.D.S.) underwent staged implantation of deep brain stimulation (DBS) leads as part of their treatment ^18,19^. Prior to surgery, written informed consent was obtained from patients or their legal guardians (for minors), in compliance with HIPAA regulations, authorizing the use of electrophysiological data for research purposes. The study protocols received approval from the institutional review boards (IRBs) at Children’s Hospital of Orange County, Rady Children’s Health, and Children’s Hospital Los Angeles (CHLA). All methods were performed in accordance with the relevant guidelines and regulations. Demographic information for the participants is detailed in Table 1. The majority of patients presented with generalized dystonia. A subset exhibited regionally predominant symptoms (e.g., orofacial in S5, oromandibular and lower limbs in S7, and arm-dominant in S13).

**Table 1:** Demographic and recording information for all participants included in this study.

Subject	Etiology	Type	Affected body site	No. GPi recordings	No. STN recordings	No. VO recordings
S1	MEPAN	Secondary	Generalized	6	4	0
S2	MEPAN	Secondary	Generalized	2	6	1
S3	KMT2B	Primary	Generalized	0	0	2
S4	GA1	Secondary	Generalized	14	12	3
S5	Atypical PKAN	Secondary	Orofacial	28	12	2
S6	HIE	Secondary	Generalized	5	10	2
S7	MYH2	Secondary	Oromandibular + lower limbs	16	3	0
S8	CP (Prematurity)	Secondary	Generalized	0	1	0
S9	GA1	Secondary	Generalized	1	4	1
S10	Kernicterus ^27^	Secondary	Generalized	13	7	1
S11	BG hemorrhage	Secondary	Generalized	0	0	0
S12	KMT2B	Primary	Generalized	29	21	5
S13	HIE	Secondary	Generalized (arms-dominant)	17	14	8

The table details the patients’ etiology, dystonia type, affected body site and the number of recording samples in each region (GPi, STN, VO) that exhibited EPs at 55 Hz. Only correctly placed recordings where EPs were detected at 55 Hz were included in further analyses. All subjects were male, aged between 5 and 24 years. Abbreviations: MEPAN: Mitochondrial Enoyl CoA Reductase Protein-Associated Neurodegeneration^20^, KMT2B: Lysine Methyltransferase-2B^21^, GA1: Glutaric aciduria type 1^22^, PKAN: Pantothenate Kinase-Associated Neurodegeneration^23^, HIE: Hypoxic-ischemic encephalopathy^24^, MYH2: myosin heavy chain IIa gene^25^, CP: Cerebral palsy^26^, BG: Basal Ganglia.

### DBS implantation and electrophysiological recording

**Fig. 1:**
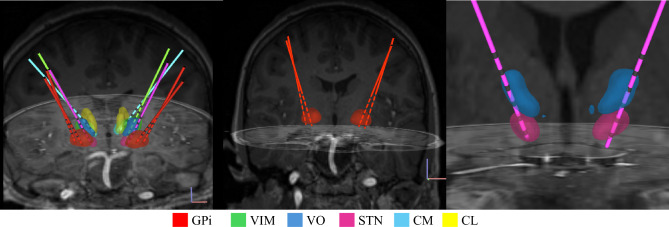
Postoperative localization of implanted DBS leads based on co-registered MRI and CT images for subject S13. The left panel displays all implanted leads and their target regions. For this subject, five leads were implanted in each hemisphere: two in GPi (red), one in VIM (green), one passing through VO (dark blue) and STN (pink), and one spanning CM (light blue) and CL (yellow). The middle panel highlights the GPi region (red), while the right panel focuses on the VO (blue) and STN (pink) regions. These neuroimaging-based localizations guided the selection of recording contacts for EP detection.

AdTech MM16C DBS leads (Adtech Medical Instrument Corp., Oak Creek, WI, USA) were surgically implanted bilaterally in each patient at various potential brain targets, including the STN, GPi in the basal ganglia, ventral intermediate nucleus (VIM), VO, ventral anterior nucleus (VA), centromedian nucleus (CM), and central lateral nucleus (CL) in the thalamus, as well as the pedunculopontine nucleus (PPN) and substantia nigra reticulata (SNr) in the brainstem. The selection of these sites was primarily based on the patient’s specific symptoms and diagnosis by utilizing a standard stereotactic approach^18,19^. These targets were chosen due to their established clinical efficacy in DBS based on existing literature^28^. The validation of lead positioning was assessed postoperatively by co-registering preoperative magnetic resonance imaging (MRI) with postoperative computed tomography (CT) scans. An example of these leads, localized based on neuroimaging, can be seen in Fig. 1.

Each stereoelectroencephalography (sEEG) lead consisted of ten high-impedance (70–90 k$\Omega$) micro-wire electrodes, each with a diameter of 50 $\upmu$m, and six low-impedance (1–2 k$\Omega$) ring macro-contacts. The macro-contacts were 2 mm in height and spaced 5 mm apart. The leads were connected to a recording device using Adtech Cabrio$^\text {TM}$ connectors equipped with preamplifiers to reduce signal noise. Stimulation was delivered through the macro-contacts, while electrophysiological data were recorded from the micro-contacts at a sampling rate of approximately 24 kHz using a customized recording system (TDT, Tucker-Davis Technologies Inc., Alachua, FL, USA).

### Stimulation protocol

The study was conducted 2 to 3 days after the implantation of temporary DBS leads. The experimental protocol, based on a similar approach described in^29^, aimed to investigate the impact of different DBS frequencies commonly used in dystonia treatment. A frequency sweep was performed at 55, 85, 185, and 250 Hz across all adjacent macro-contact pairs on each implanted lead. For each frequency-contact pair, stimulation was applied twice: first with one contact serving as the cathode and the adjacent contact as the anode, and then with the polarity reversed such that the previously anodic contact became the cathode and vice versa. In each polarity condition, stimulation was delivered at a target of approximately 1000 pulses, with each pulse lasting 90 µs. To account for potential signal noise and artifact detection errors during preprocessing, we collected slightly more than 1000 pulses per condition. This allowed us to retain exactly 1000 clean, artifact-aligned segments for analysis after quality control. The default stimulation voltage was set at 3 V, but if the patient experienced discomfort, it was reduced to a more tolerable level. For frequencies $\ge 185$ Hz, a voltage ramping protocol was applied, gradually increasing the voltage from 0 to 3 V over 12 seconds to improve patient comfort. As a result, the total recording duration exceeded the minimum required for 1000 pulses and varied with frequency: approximately 25 seconds for 55 Hz, 15 seconds for 85 Hz, 35 seconds for 185 Hz, and 30 seconds for 250 Hz. Throughout these stimulations, intracranial signals were recorded continuously from all leads and brain regions, ensuring comprehensive capture of DBS-evoked activity. In this study, we only investigated the effect of GPi stimulation on GPi, VO, and STN recordings. We refer to these as GPi-GPi, GPi-VO, and GPi-STN, respectively, following the convention of stimulation region–recording region.

### Electrophysiological data processing and analysis

All data processing and analysis were done in MATLAB R2023b (The MathWorks, Inc., Natick, MA, USA).

#### Preprocessing of neural signals

The LFP signals underwent notch filtering at 60 Hz and its first five harmonics, followed by high-pass filtering at 1 Hz to remove low-frequency drift. To enhance signal quality, bipolar re-referencing was applied by subtracting signals from adjacent micro-contact pairs on each lead, producing 8 channels per lead. For instance, instead of analyzing micro-contacts individually, voltage differences between contact pairs (e.g., 1-2, 1-3, and 2-3) were used. This approach reduces common noise and enhances the detection of neural activity.

The bipolar recordings were then upsampled to 120 kHz, and stimulus artifact peaks were identified using MATLAB’s ‘findpeaks’ function. The signals were segmented into 11-ms windows, starting 1 ms before the stimulus artifact onset. Non-outlier segments were aligned via cross-correlation of time-0 artifacts, yielding approximately 1000 segments per stimulus location. Finally, these segments were averaged to improve the signal-to-noise ratio (SNR), following the stimulus-triggered averaging method proposed by Sinclair et al. ^30^. This process was conducted independently for recordings obtained during both stimulation polarities at each contact pair. The resulting signals from both polarities were then combined to generate a polarity-reversed average, which substantially reduces the stimulation artifact, improving the detectability of the underlying evoked response. However, particularly in GPi recordings where stimulation and recording contacts are in close proximity, some residual artifact components may persist. ^31–33^.

#### EP detection algorithm

**Fig. 2:**
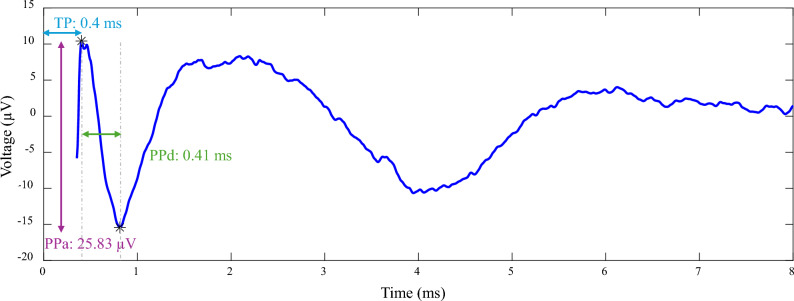
Example of an EP from subject S13 (GPi-GPi), illustrating key EP characteristics: peak-to-peak amplitude (PPa), peak-to-peak duration (PPd), and time to peak (TP). The waveform shown is a polarity-reversed average, where the decay artifact is minimized and the stimulation artifact is blanked out.

After processing the data and averaging the cathodic and anodic segments, decay artifacts were removed using the method described by Vidmark et al. ^31^. Figure 2 presents an example of a polarity-reversed average, where the decay artifact is minimized and the stimulation artifact is blanked out. Following artifact removal, an automated algorithm called ADPREP was applied to detect EPs ^34^. The algorithm operates on the principle that a neural response evoked by stimulation at a given contact pair should be similarly elicited if the polarity is reversed (i.e., cathodic vs. anodic stimulation). Thus, a true neural response should exhibit a significant positive correlation between the artifact-reduced, polarity-reversed recordings. To confirm the presence of EPs, the algorithm examines potential EP regions that display high positive correlation by detecting positive and negative peaks. The region with the highest peak-to-peak amplitude (PPa) is then identified as the primary neural response.

Finally, the PPa amplitude is compared against a user-defined minimum amplitude threshold, ensuring a sufficient SNR (threshold = 7, determined via visual inspection). If the PPa surpasses this threshold, the detected peaks are classified as an EP. Otherwise, the recording is labeled as not containing an EP.

#### Contact selection and recording pruning

Since this study focuses on recordings from GPi, VO, and STN, the EP detection pipeline was applied only to contact pairs located within these regions, as determined by neuroimaging. For instance, as shown in Fig. 1, in a lead passing through VO and STN in the left hemisphere, contacts in row 2 and 3 were classified as STN, while contacts in row 4 were assigned to VO. For all subjects, in both hemispheres, contacts located in GPi, VO, and STN were visually identified using neuroimaging data. Following this, an additional contact pruning step was performed.

To refine the dataset, EP detection was initially conducted on correctly placed recordings at 55 Hz, which was considered the baseline condition. If no EP was detected at 55 Hz using the algorithm, those recordings were excluded from the study, and EP detection was not attempted for those contacts at other stimulation frequencies. Table 1 summarizes the number of recordings that exhibited EPs at 55 Hz for each subject in each region. As shown in the table, subject S11 had no recordings with detectable EPs in any region and was excluded from further analyses. This was not due to hardware failure or lead misplacement; postoperative imaging confirmed proper targeting. Rather, it is likely attributable to individual variability in EP expression, signal-to-noise ratio, or network responsiveness, as the 55 Hz EPs did not meet the detection threshold defined by ADPREP.

For the remaining contacts, the time of the positive and negative EP peaks at 55 Hz stimulation was determined. EP detection at other stimulation frequencies was then constrained to occur within the same time window as the baseline condition (55 Hz). This time-constrained approach ensured greater consistency in EP detection across different conditions, facilitating more reliable comparisons.

#### EP characterization

For all detected EPs across recordings, three measures were used to characterize their morphology:

- Peak-to-Peak Amplitude (PPa), defined as the amplitude difference between the positive and negative peaks.
- Peak-to-Peak Duration (PPd), representing the time interval between the positive and negative peaks.
- Time to Peak (TP), which is the time from the onset of the stimulation artifact to the occurrence of the first peak.

For group-level analyses, these measures were normalized to their respective values at the baseline condition of 55 Hz. This normalization was performed to account for inter-subject variability in absolute EP amplitudes (which can range from approximately 10 to 100 $\mu$V) and to more clearly reveal underlying trends across stimulation conditions. Note that this normalization was applied only for group analyses, while individual examples are presented using their raw values. An example illustrating these characteristics in an EP is shown in Fig. 2.

### Statistical analysis

All statistical analyses were performed in RStudio 2024.04 (Posit, PBC, Boston, MA, USA) using R version 4.3.2 (R Foundation for Statistical Computing, Vienna, Austria).

To examine the effect of stimulation frequency on EP characteristics within each recording region, we employed a linear mixed effects (LME) model, incorporating subject ID as a random variable to account for inter-subject variability. The model was formulated as Equation 1 and implemented using the ‘lmerTest::lmer’ function with the maximum likelihood estimation method. Post-hoc pairwise comparisons between stimulation frequencies were conducted using the ‘emmeans’ package, with Bonferroni corrections applied to adjust for multiple comparisons.

To assess differences in the frequency-dependent trends of EP measures across recording regions, we first applied a generalized linear model (GLM) to estimate the slope of the trend in each region. Subsequently, another LME model was used on the full dataset (all three regions combined) to investigate the interaction effect between recording region and stimulation frequency. This model was formulated as Equation 2.

1 $$\begin{aligned}&\text {EP measure} \sim \text {Stimulation Frequency} + (1 | \text {ID}) \end{aligned}$$

2 $$\begin{aligned}&\quad \text {EP measure} \sim \text {Stimulation Frequency} \text { * Recording Region} + (1 | \text {ID}) \end{aligned}$$

## Results

### Frequency-dependent changes in EP measures

**Fig. 3:**
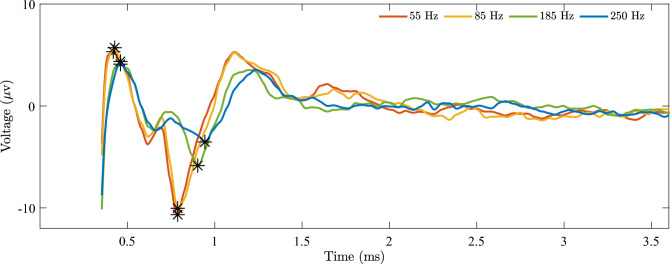
Detected changes in EP characteristics across different stimulation frequencies. With increasing stimulation frequency, PPa decreases, PPd increases, and TP shows a slight increasing trend. For 55, 85, 185, and 250 Hz stimulation, PPa values are 15.99, 15.75, 9.96, and 7.9 $\mu$V; PPd values are 0.37, 0.36, 0.44, and 0.48 ms; and TP values are 0.42, 0.43, 0.46, and 0.46 ms, respectively. Data shown is from subject S10 with GPi stimulation and GPi recording (GPi-GPi condition).

We first examined the effect of stimulation frequency on EP characteristics across all recording regions. A representative example of EP waveforms from a single subject (GPi stimulation, GPi recording: GPi-GPi) is shown in Fig. 3. Across increasing stimulation frequencies (55, 85, 185, and 250 Hz), we observed a decrease in PPa (15.99, 15.75, 9.96, and 7.9 $\mu$V, respectively) and an increase in PPd (0.37, 0.36, 0.44, and 0.48 ms, respectively), while TP exhibited a mostly increasing trend (0.42, 0.43, 0.46, and 0.46 ms, respectively).

**Fig. 4:**
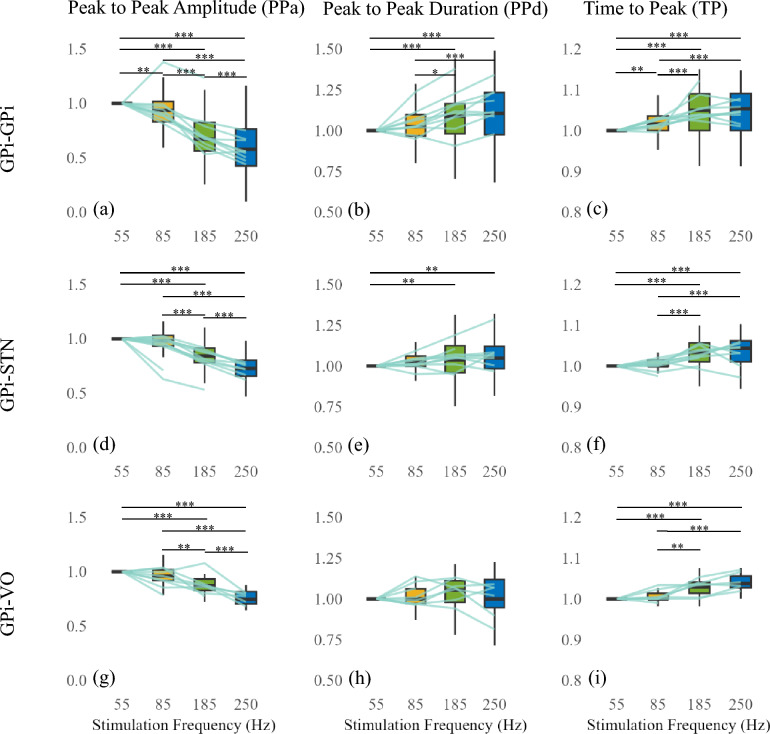
Effect of different stimulation frequencies (55, 85, 185, 250 Hz) on various EP characteristics. Columns represent normalized EP characteristics: PPa, PPd, and TP, in that order. All graphs within the same column share the same scale for comparability across recording regions. Rows represent different recording regions: Row 1 (a, b, c) shows the effect of GPi stimulation recorded in GPi, Row 2 (d, e, f) represents VO recordings during GPi stimulation, and Row 3 (g, h, i) displays STN recordings in response to GPi stimulation. Subject-specific data are visualized with gray lines connecting the average EP of each subject. Statistical significance is indicated by asterisks: * for $p < 0.05$, ** for $p < 0.01$, and *** for $p < 0.001$, connecting relevant box plots. No line is used when there was no significance between the two box plots.

To assess whether these trends were consistent across subjects and regions, we performed group-level analyses with PPa, PPd, and TP normalized to 55 Hz values (Fig. 4). Across all recording sites, PPa significantly decreased while PPd and TP increased with frequency. However, statistical analysis revealed a notable threshold effect, where 55 Hz and 85 Hz were not significantly different, but stronger modulation emerged at $\ge$ 185 Hz. We use the term threshold effect here to refer to the sharp increase in EP attenuation and temporal broadening observed between 85 Hz and 185 Hz, rather than to a clinical stimulation threshold or symptom response. We used an LME model (Equation 1) incorporating subject ID as a random effect to account for inter-subject variability. Bonferroni-corrected pairwise comparisons confirmed that most significant differences occurred between 85 Hz and 185 Hz, rather than between 55 Hz and 85 Hz. This suggests a potential transition point where frequency-dependent effects become more pronounced.

Despite some inter-subject variability (visualized with gray lines in Fig. 4), most subjects followed the same overall pattern, confirming the robustness of these trends.

### Regional variability in frequency-dependent EP changes

**Fig. 5:**
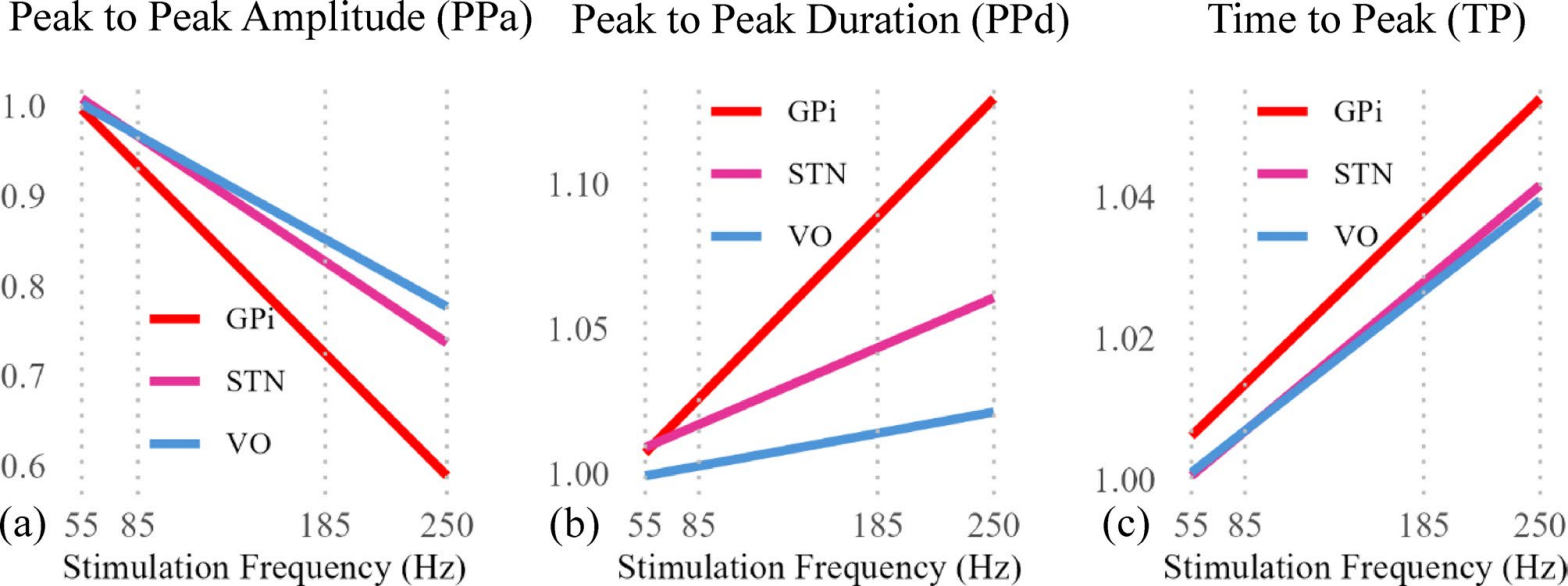
Effect of stimulation frequency on EP characteristics across recording regions. The slopes from the GLM illustrate the relationship between stimulation frequency and (a) PPa, (b) PPd, and (c) TP. All measures are normalized to their values at 55 Hz. Across all metrics, GPi (red) exhibits the steepest slopes, indicating a stronger modulation of EP characteristics by stimulation frequency compared to STN (pink) and VO (blue). The weakest frequency-dependent effect is observed for PPd in VO.

Although similar trends were observed across all regions, their magnitude varied by recording location. To quantify these regional differences, we applied a GLM model to assess the slope of frequency-dependent changes in each region (Fig. 5). GPi recordings exhibited the steepest slopes, indicating stronger frequency-dependent modulation compared to STN and VO.

Statistical analysis confirmed that stimulation frequency had a significant effect on PPa and TP in all regions (p < 0.001). However, for PPd, the effect was significant in GPi-GPi and GPi-STN (p < 0.001), but not in GPi-VO (p = 0.396) (Table 2), suggesting that EP duration is less sensitive to stimulation frequency in VO.

**Table 2:** GLM results for the effect of stimulation frequency on normalized EP characteristics across different recording regions. Regression coefficients ($\beta$), standard errors (SE), t-values, and p-values are reported. NS stands for non significant.

EP Measure	Region	$\beta$ (Estimate)	SE	t-value	p-value
PPa	GPi-GPi	-0.00208	0.00010	-20.32	<0.001 ***
	GPi-STN	-0.00139	0.00006	-19.93	<0.001 ***
	GPi-VO	-0.00115	0.00011	-9.97	<0.001 ***
PPd	GPi-GPi	0.00062	0.00009	6.45	<0.001 ***
	GPi-STN	0.00026	0.00006	3.89	<0.001 ***
	GPi-VO	0.00011	0.00013	0.85	0.396 (NS)
TP	GPi-GPi	0.00024	0.00002	9.65	<0.001 ***
	GPi-STN	0.00021	0.00002	10.35	<0.001 ***
	GPi-VO	0.00019	0.00002	7.37	<0.001 ***

**Fig. 6:**
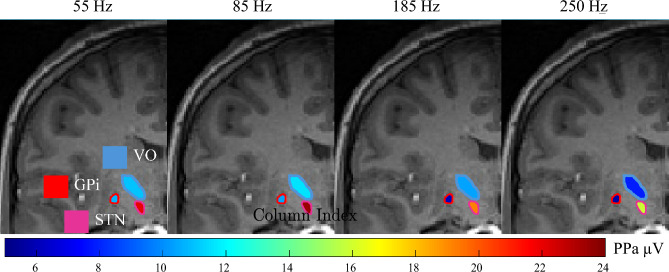
The propagation of GPi stimulation in the GPi (red outline), VO (blue outline), and STN (pink outline) regions across different stimulation frequencies. The fill colors represent the PPa of EPs detected in these regions. VO and GPi PPa start at approximately 10 $\mu V$ at 55 Hz and gradually decrease to around 6 $\mu V$ as the stimulation frequency increases, becoming darker in color. In STN, the initial PPa is red, gets darker at 85 Hz, and then shifts back to orange and yellow with further increase in stimulation frequency. The MRI image and region localization is estimated based on neuroimaging data from subject S13.

Figure 6 visually represents these regional differences using a single-subject colormap of PPa amplitudes at different stimulation frequencies. Across all regions, PPa decreases with increasing frequency, but the rate of this decrease varies: GPi and VO exhibit an initial PPa of around 10 $\mu$V at 55 Hz, which declines to almost 6 $\mu$V at 250 Hz, whereas STN starts at a higher PPa and follows a different decay pattern. These findings highlight that the optimal stimulation frequency may need to be tailored to the target region, as different structures exhibit distinct responses to frequency modulation.

Finally, to determine whether the effect of stimulation frequency was statistically different between regions, we applied the LME model (Equation 2), testing for interaction effects between stimulation frequency and recording region. The results (Table 3) showed that the effect of stimulation frequency on PPa was significantly different between GPi and STN ($\beta$ = 0.00069, p < 0.001) as well as GPi and VO ($\beta$ = 0.00093, p < 0.001). A similar but weaker effect was observed for PPd, where the frequency effect differed significantly between GPi and STN ($\beta$ = -0.00034, p = 0.005) as well as VO ($\beta$ = -0.00049, p = 0.014). However, for TP, the interaction terms were not significant (STN: p = 0.299; VO: p = 0.363), indicating that the effect of stimulation frequency on TP did not differ significantly across recording locations.

**Table 3:** Results of the LME model examining the interaction between stimulation frequency (“Stim Freq”) and recording location on normalized EP characteristics: PPa, PPd, and TP. Only interaction effects are reported, with GPi serving as the baseline recording region. NS indicates non-significant results.

EP Measure	Predictor	$\beta$ (Estimate)	SE	t-value	p-value
PPa	Stim Freq $\times$ STN Recording	0.00069	0.00012	5.33	<0.001 ***
	Stim Freq $\times$ VO Recording	0.00093	0.00020	4.51	<0.001 ***
PPd	Stim Freq $\times$ STN Recording	-0.00034	0.00012	-2.79	0.005 **
	Stim Freq $\times$ VO Recording	-0.00049	0.00019	-2.46	0.014 *
TP	Stim Freq $\times$ STN Recording	-0.00003	0.00003	-1.03	0.299 (NS)
	Stim Freq $\times$ VO Recording	-0.00004	0.00005	-0.91	0.363 (NS)

## Discussion

This study investigated how different stimulation frequencies applied to the GPi, modulate EP characteristics within the basal ganglia-thalamo-cortical network in pediatric dystonia patients. We found that increasing stimulation frequency systematically decreased PPa and increased PPd and TP, with a clear distinction between low-frequency (55, 85 Hz) and high-frequency (185, 250 Hz) stimulation. Furthermore, these effects were most pronounced locally at the GPi recording site compared to STN and VO, highlighting that the impact of DBS frequency varies depending on the recording region.

Our findings align with previous DBS-evoked potential studies that have demonstrated frequency-dependent modulation of neural responses across multiple brain disorders. For example, cortical evoked potentials (cEPs) have been extensively studied^13,35–39^ and their characteristics have shown to be frequency dependent during STN-DBS^35^. In PD, short-latency cEPs generated by STN-DBS have been shown to reduce in amplitude and increase in latency as stimulation frequency rises^13^. In healthy rats, Kumaravelu et al.^40^ similarly reported that during STN-DBS, the shortest-latency EP component reached minimal amplitude and maximal latency at 130 Hz compared to lower frequencies. These frequency-dependent EP changes appear to reflect a fundamental property of neural circuits under rapid stimulation: as pulse frequency increases, the neural response becomes smaller and slower, likely due to cumulative refractoriness and short-term synaptic depression. This includes mechanisms such as presynaptic vesicle depletion and reduced neurotransmitter release, which limit synaptic efficacy at high stimulation rates^41,42^.

Liu et al.^14^ examined various microstimulation frequencies, ranging from 1 to 100 Hz in a group of 13 dystonia patients to examine the characteristics of EPs. Their results demonstrated an increase in PPa from 1 to 30 Hz followed by a declined in PPa for higher frequencies. Our results support and extend their findings where GPi-DBS at frequencies higher than 50 Hz continues to dampen the EP amplitudes. Taken together, these findings suggest that neural responsiveness saturates or enters a refractory pattern at high stimulation rates.

Furthermore, our observation of regional differences (GPi > STN > VO in effect magnitude) is supported by prior electrophysiological studies. Schmidt et al.^15^ simultaneously recorded STN and GP evoked responses during DBS and found that the STN-GP circuits are highly interlinked, with EP characteristics tied to intrinsic network oscillations. Our data suggest that the pallidal circuit may be particularly sensitive to high-frequency input, possibly due to its dense inhibitory connections and integrative role as an output nucleus. The weaker effects in VO align with findings by Awad et al.^43^, who demonstrated that short-term plasticity of EPs differs by target: paired-pulse DBS facilitated responses in STN and GPi recordings but not in thalamic recordings.

It is also important to consider that the weaker frequency-dependent modulation observed in VO may reflect not only intrinsic circuit properties but also spatial distance from the stimulation site. In some subjects, EP amplitudes in VO and GPi were comparable at low frequencies (e.g., 55 Hz), but VO showed less attenuation in amplitude with increasing frequency. This suggests that anatomical separation may reduce the degree to which stimulation modulates VO responses, either by limiting current spread or by dampening the propagation of evoked activity. These spatial considerations, in combination with differences in thalamic versus basal ganglia circuitry, likely contribute to the regional variability in EP modulation. Together, these findings highlight that DBS effects are not uniformly distributed across targets and reinforce the stronger frequency sensitivity of basal ganglia structures compared to thalamic nuclei.

### Neurophysiological implications

Our results contribute to a broader understanding of how DBS frequency modulates basal ganglia network activity. The frequency-dependent reduction in PPa likely reflects activity-dependent saturation or inhibition within the local circuit. High-frequency DBS (>100 Hz) delivers pulses at intervals shorter than synaptic recovery times, leading to progressive recruitment failure or reduced neurotransmitter release^44,45^. On the other hand, our result is consistent with the hypothesis that high-frequency GPi-DBS induces inhibitory effects via increased GABAergic output^46^, which may contribute to the suppression of pathological oscillations in dystonia^47^. While inhibitory mechanisms—such as increased GABAergic output and synaptic suppression—likely contribute to the attenuation of EPs, DBS has also been shown to exert excitatory and modulatory effects in certain contexts. These include activation of excitatory afferents, antidromic activation of cortical projections, and modulation of thalamocortical relay neurons^44,48,49^. Thus, the net effect of DBS likely reflects a dynamic interplay between excitatory and inhibitory influences, varying by target region and frequency.

The increase in PPd and TP suggests that as stimulation frequency rises, neural transmission through polysynaptic pathways or slower conduction fibers becomes more prominent. Similar latency shifts have been observed in cortical EPs recorded in response to STN-DBS in PD, where the fastest responses diminish at high frequencies, and only slower, polysynaptic pathways remain active^13^. This finding suggests that DBS frequency not only influences direct excitation but also alters network dynamics by shifting the balance between fast and slow circuit components.

The threshold effect observed at $\ge$185 Hz, where frequency-dependent changes in PPa, PPd, and TP became more pronounced, indicates that DBS may engage distinct neural mechanisms above this frequency. Previous research has suggested that stimulation at lower frequencies may allow for discrete neuronal responses, while higher frequencies drive continuous network activity, potentially overriding pathological oscillations^15^. Our findings provide evidence that crossing a certain frequency threshold induces a qualitative shift in neural recruitment, possibly linked to increased entrainment of inhibitory interneurons or a switch in dominant synaptic mechanisms.

Additionally, the regional differences in EP modulation (GPi > STN > VO) reinforce the idea that different brain structures respond uniquely to DBS frequency due to their distinct neuroanatomical properties. The GPi, as an output nucleus of the basal ganglia, integrates inhibitory signals from the striatum and excitatory inputs from the STN, making it particularly sensitive to frequency-dependent effects^50,51^. STN, as a key excitatory driver ^52^, exhibits intermediate sensitivity, whereas thalamic VO recordings show the weakest modulation, likely due to its role as a relay nucleus with less recurrent inhibition ^53^.

### Clinical implications for dystonia treatment

Although our study does not establish a direct link between neurophysiological changes and clinical efficacy, our findings have important implications for personalized DBS programming in dystonia. The fact that frequency-dependent changes in EPs were region-specific suggests that optimal stimulation settings should be tailored to the target region rather than applying a one-size-fits-all approach.

Current DBS programming typically starts at 130 Hz and is adjusted based on clinical response^8^. Our results suggest that for GPi-targeted DBS, higher frequencies (185–250 Hz) produce stronger physiological modulation, while lower frequencies (55–85 Hz) evoke smaller changes in EP morphology. These findings are particularly relevant given that some dystonia patients require higher stimulation frequencies (>150 Hz) for optimal symptom relief, whereas others achieve benefit at lower settings^16,17^. We do not suggest that lower frequencies are clinically ineffective. Rather, clinical efficacy likely depends on additional factors such as etiology, circuit excitability, and electrode positioning. This variability underscores the importance of a personalized approach to frequency selection and highlights the need to further investigate how EP modulation relates to therapeutic outcomes.

Additionally, our findings contribute to the growing interest in DBS-evoked potentials as potential biomarkers for optimizing stimulation settings^8,43^. If specific EP characteristics (e.g., the degree of PPa attenuation or the extent of TP prolongation) correlate with clinical outcomes, they could be used to refine programming decisions, potentially improving clinical efficacy while minimizing energy consumption. Notably, the rate of EP attenuation differed across recording sites, suggesting that DBS frequency may not only modulate local activity but also shape the distribution of its effects across the basal ganglia-thalamo-cortical network. This raises the possibility that frequency adjustments could be used to fine-tune how stimulation propagates to different regions, offering an additional dimension for personalized DBS optimization. In clinical practice, this could involve selecting contacts nearest to the region that shows the most physiologically robust or clinically beneficial response to specific frequencies. Testing a range of frequencies while monitoring local EPs may inform target-specific programming. Furthermore, as adaptive DBS systems evolve, regionally-informed biomarkers such as EP amplitude or latency could be used to guide dynamic frequency modulation in real time.

### Limitations and future directions

While this study provides valuable insights into frequency-dependent EP changes in dystonia, several limitations should be acknowledged:

Our sample consisted of 13 pediatric and young adult dystonia patients, all of whom were male. The cohort included both primary (only 2 patients) and secondary dystonia cases with varying clinical presentations. Although consistent frequency-dependent trends were observed, this homogeneity in sex and heterogeneity in etiology may limit generalizability. Future studies should include more diverse populations and stratify analyses by dystonia subtype to determine whether EP responses vary across clinical subgroups.

Although group-level trends were robust, individual variability in EP amplitude and latency was substantial. This may stem from differences in electrode placement, impedance, and underlying neurophysiology. Moreover, we did not collect concurrent clinical outcome measures at each frequency, limiting our ability to link EP features directly to symptom improvement. Future work should incorporate clinical assessments and explore whether EP dynamics correlate with treatment efficacy on a patient-specific basis.

All recordings were obtained in the acute post-implantation phase, prior to chronic DBS. While some physiological effects of stimulation can emerge within seconds to minutes ^54^—particularly in thalamic and subthalamic regions—dystonia symptom relief often unfolds over weeks or months. Thus, our results may not fully reflect chronic stimulation effects. Longitudinal studies using sensing-enabled systems (e.g., Medtronic Percept PC) could help track long-term changes in EPs and their clinical relevance.

We tested discrete frequencies up to 250 Hz, but finer-grained increments (e.g., 100–160 Hz) would help clarify whether the observed threshold-like transition between 85 and 185 Hz is abrupt or gradual. Future studies should also investigate ultra-high-frequency and patterned DBS paradigms, which may elicit distinct network effects. Incorporating machine learning tools for EP analysis and exploring real-time EP-guided adaptive stimulation could ultimately support personalized, closed-loop DBS programming strategies.

## Conclusion

Our study demonstrates that DBS frequency strongly modulates EP characteristics in dystonia, with distinct effects depending on the recording region. The findings highlight a clear distinction between low (55–85 Hz) and high (185–250 Hz) frequency stimulation, with the most pronounced effects observed locally in GPi recordings. These results underscore the need for personalized DBS programming, where frequency tuning is tailored to the target region. While further research is needed to establish clinical correlations, our findings provide a neurophysiological basis for optimizing DBS parameters in dystonia treatment.

## Data Availability

The data analyzed in this study is subject to the following licenses/restrictions: The datasets were collected as part of the DBS procedure for clinical and research purposes. They are considered protected health information under HIPAA. De-identified datasets may be available on request. Requests to access these datasets should be directed to Terence Sanger, terry@sangerlab.net.
